# Tumor resection ameliorates tumor-induced suppression of neuroinflammatory and behavioral responses to an immune challenge in a cancer survivor model

**DOI:** 10.1038/s41598-018-37334-8

**Published:** 2019-01-24

**Authors:** Jessica C. Santos, Savannah R. Bever, Gabriela Pereira-da-Silva, Leah M. Pyter

**Affiliations:** 1 Institute for Behavioral Medicine Research, Ohio State University Wexner Medical Center, Columbus, OH USA; 2 Postgraduate Program in Basic and Applied Immunology, Ribeirão Preto School of Medicine, University of São Paulo, Ribeirão Preto, SP Brazil; 3 Department of Psychiatry and Behavioral Health, Ohio State University, Columbus, OH USA; 4 Department of Maternal-Infant Nursing and Public Health, Ribeirão Preto College of Nursing, University of São Paulo, Ribeirão Preto, SP Brazil; 5 Department of Neuroscience, Ohio State University, Columbus, OH USA; 6 Arthur G. James Comprehensive Cancer Center and Solove Research institute, Ohio State University, Columbus, OH USA

## Abstract

Breast cancer survivors display altered inflammatory responses to immune challenges relative to cancer-naive controls likely due to previous cancer treatments, stress associated with cancer, and/or tumor physiology. Proper inflammatory responses are necessary for adaptive sickness behaviors (e.g., fatigue, anorexia, and fever) and neuroinflammatory pathways are also implicated in mental health disturbances (e.g., cognitive impairment, depression) suffered by cancer patients and survivors. Rodent cancer models indicate that tumors are sufficient to exacerbate neuroinflammatory responses after an immune challenge, however primary tumors are not usually present in cancer survivors, and the behavioral consequences of these brain changes remain understudied. Therefore, we tested the extent to which mammary tumor resection attenuates tumor-induced neuroinflammation and sickness behavior following an immune challenge (i.p. lipopolysaccharide [LPS] injection) in mice. *Tnf*-*α*, *Il*-*1β*, and *Il*-*6* mRNA decreased in multiple brain regions of LPS-treated tumor-bearing mice relative to LPS-treated controls; tumor resection attenuated these effects in some cases (but not *Tnf*-*α*). Tumors also attenuated sickness behaviors (hypothermia and lethargy) compared to LPS-treated controls. Tumor resection reversed these behavioral consequences, although basal body temperature remained elevated, comparable to tumor-bearing mice. Thus, tumors significantly modulate neuroinflammatory pathways with functional consequences and tumor resection mitigates most, but not all, of these changes.

## Introduction

Cancer patients receiving treatment and survivors that have completed treatment, as well as rodent tumor models, exhibit elevated inflammatory profiles at rest that correlate with negative behavioral comorbidities^1–8^ (but see refs^9,10^). Furthermore, both cancer patients and survivors display altered physiological responses to acute physiological and psychological challenges^11^. Challenging a physiological system allows for the assessment of underlying pathway function, as opposed to assessing resting, baseline physiology. Persistently altered immune responsivity to daily common challenges (e.g., stress, influenza), for example, may additively contribute to the debilitating quality of life issues typical of cancer survivors^12^. Indeed, peripheral immune activation drives immune activation in the brain and is strongly implicated in both sickness behaviors (e.g., fatigue) and psychiatric conditions relevant to cancer survivors^13^, including depression and cognitive impairments^14^. While chemotherapy is often assumed to be the cause of such behavioral consequences in cancer populations (“chemobrain”), evidence indicates that these comorbidities can arise prior to cancer treatments^15,16^, suggesting a causal role of tumor-associated inflammation or other tumor biology. Indeed, non-CNS tumors alone cause mild basal neuroinflammatory and behavioral changes in rodent cancer models^4,8^, similar to neuroinflammatory “priming” observed in other health contexts (e.g., chronic stress)^17^. In these contexts, priming of brain immune cells can lower the threshold for subsequent activation of inflammatory pathways resulting in exaggerated or pathological neuroinflammation and negative behavioral consequences^18^.

In the cancer field, the majority of the clinical research testing this “two-hit” hypothesis has focused on endocrine responses to acute stressors^11^. However, more recent work examines immune responses. Studies using acute psychological stressors to probe peripheral cytokine or leukocyte responses among subsets of cancer survivors (fatigued, smokers) intimate that cytokine responsivity may be somewhat increased or delayed among cancer survivors^19,20^. In contrast, two studies in current cancer patients have used immune challenges and observed either no alterations in cytokine responses (after interferon-α administration^21^) or elevated cytokine responses (after surgery^22^). Thus, alterations in inflammatory responses relevant to behavior may vary between cancer patients and survivors.

As a first step in understanding how tumors may alter immune responses in the brain (as opposed to at rest), our previous study compared neuroinflammatory responses to an acute peripheral challenge between tumor-bearing and tumor-free rats. This study indicated that carcinogen-induced mammary tumors exacerbate neuroinflammatory responses to a peripheral infection mimetic (lipopolysaccharide, LPS)^4^. Corroborating evidence of select exacerbated neuroinflammatory markers and sickness behavior is observed in a murine head and neck tumor model following LPS administration^8^. To advance this work, the current study aimed to determine: (1) the extent to which these neuroinflammatory changes are attenuated by tumor resection in a novel mouse non-metastatic breast cancer survivor model, and (2) the potential functional consequences of altered neuroinflammation on sickness behaviors. Understanding the extent to which neuroimmune responsivity may be altered by tumor biology has potential relevance to the mental and physical health of cancer patients and survivors.

## Results

### Tumors suppress adaptive sickness behaviors

**Figure 1:**
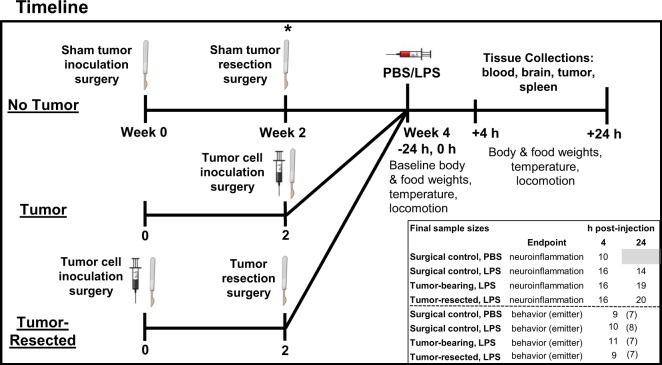
Experimental timeline and sample sizes for tumor-free (surgical controls), tumor-bearing, and tumor-resected groups. *Represents the time point for e-mitter implantation for a subset of mice. The syringes and scalpel drawings have been obtained from free clipart through Microsoft PowerPoint, released under CC-BY-SA 4.0 license (scalpel by Petit B, https://creativecommons.org/licenses/by-sa/4.0, from Wikimedia Commons).

**Figure 2:**
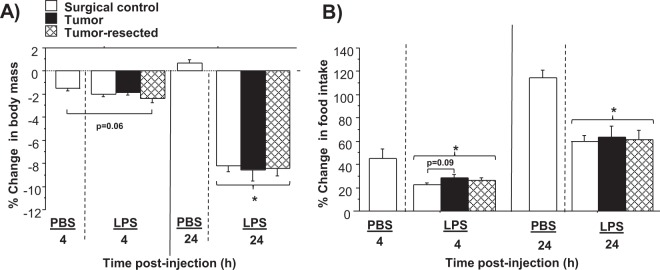
Effects of mammary tumors and tumor resection on LPS-induced sickness behaviors (anorexia). Mean (±SEM) percent change in body mass (**A**) and food intake (**B**) of surgical control, tumor-resected and –bearing mice 4 and 24 h after i.p. PBS or LPS injection relative to pre-injection baseline measures of the same timeframe (n = 9–11 mice/group). *p ≤ 0.05 LPS-treated relative to PBS-treated surgical control mice by two-way ANOVA.

The baseline measures of body mass and food intake, taken 24 h prior to the LPS injection, were not different among all groups (*p* > 0.05; see Fig. 1 for experimental design and samples size). LPS treatment significantly reduced body mass, driven primarily by the data from 24 h post-LPS (LPS main effect: *F*_1,37_ = 123.5, *p* < 0.0001; Fig. 2A). Tumor treatments did not affect changes in body mass at any time point (*p* > 0.05). LPS induced anorexia 4 h and 24 h post-LPS relative to PBS-treated controls (LPS main effect: *F*_1,37_ = 16.2, *p* < 0.0005 and *F*_1,37_ = 34.3, *p* < 0.0001, respectively; Fig. 2B). Time since LPS injection influenced food intake (time main effect: *F*_1,54_ = 54.8, *p* < 0.0001), such that anorexia receded 24 h post-LPS relative to 4 h post-LPS. Within LPS-treated mice, tumor manipulations did not alter food intake at either 4 or 24 h post-injection (*p* > 0.05), although a trend (*p* = 0.09) towards attenuated anorexia was observed in tumor-bearing mice relative to tumor-free mice at 4 h post-LPS.

**Figure 3:**
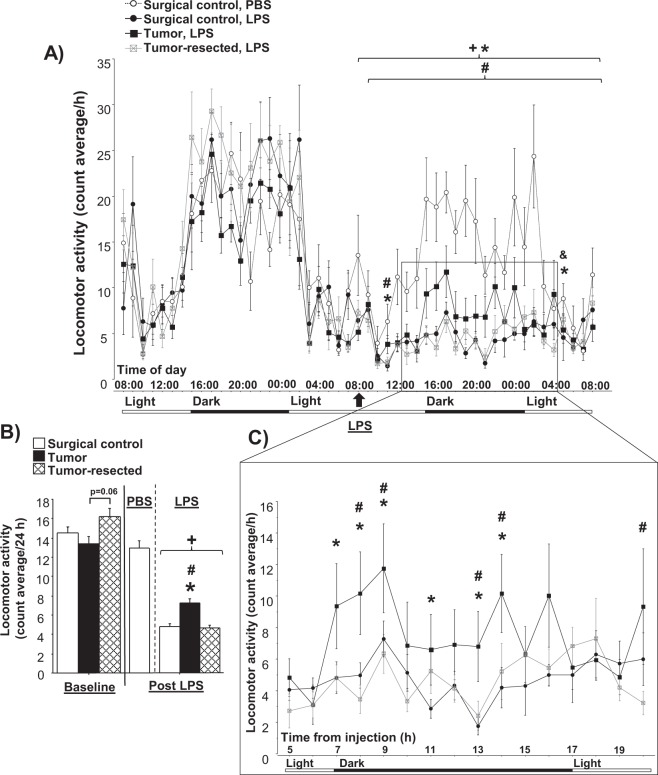
Tumor attenuation of LPS-induced lethargy is reversed after resection. (**A**) Mean (±SEM) locomotor activity (counts/h) 24 h before and 24 h after LPS injection. (**B**) Locomotor activity (average counts/24 h) in the baseline and post-LPS injection. (**C**) Increase in locomotor activity during the dark phase after LPS injection from box in (**A**). *p ≤ 0.05 tumor bearing relative to surgical control mice injected with LPS; ^#^*p* ≤ 0.05 tumor-bearing relative to –resected mice injected with LPS; ^+^*p* < 0.05 relative to the same group at the baseline; ^&^*p* ≤ 0.05 tumor-resected relative to surgical control mice injected with LPS by repeated measures ANOVA (**A**) or two-way ANOVA (**B**,**C**); (n = 6–8 mice/group).

No baseline differences in locomotion were observed among groups (*p* > 0.05; Fig. 3A and B), although a trend (*p* = 0.06) towards increased locomotion was observed in tumor-resected mice relative to tumor-bearing mice (Fig. 3B). LPS significantly decreased locomotor activity throughout the 24 h after LPS injection for all groups compared to the surgical controls injected with PBS (LPS main effect: *p* < 0.0001 for tumor-free and tumor-resected mice; *p* < 0.05 for tumor-bearing mice; Fig. 3A and B). Among LPS-treated mice, a repeated measures ANOVA yielded a main effect of tumor treatment (*F*_2,19_ = 5.5, *p* < 0.05). Although all LPS-treated mice exhibited decreased locomotor activity, tumor and time interacted to decrease the magnitude of this response in tumor-bearing mice compared to both tumor-free (*F*_1,24_ = 1.7, *p* < 0.05) and tumor-resected (*F*_1,23_ = 1.9, *p* < 0.01) mice. The LPS-induced lethargy was more apparent in the dark phase (active phase; see Fig. 3C), although decreased lethargy was observed in tumor-bearing relative to both control and -resected mice at 3 and 21 h post-LPS (tumor treatment effect: *F*_2,19_ = 6.3, *p* < 0.01 and *F*_2,19_ = 4.3, *p* < 0.05, respectively), and for tumor-resected mice relative to control mice 21 h after injection (tumor treatment effect: *F*_2,19_ = 6.3, *p* < 0.05; light phase; see Fig. 3A). During the dark phase, tumors attenuated LPS-induced reductions in locomotion relative to tumor-free (at 7, 8, 9, 11, 13 and 14 h after LPS injection) and tumor-resected mice (at 8, 9, 13 and 14 h after LPS injection; tumor treatment effect: *p* < 0.05 in all cases; Fig. 3C).

**Figure 4:**
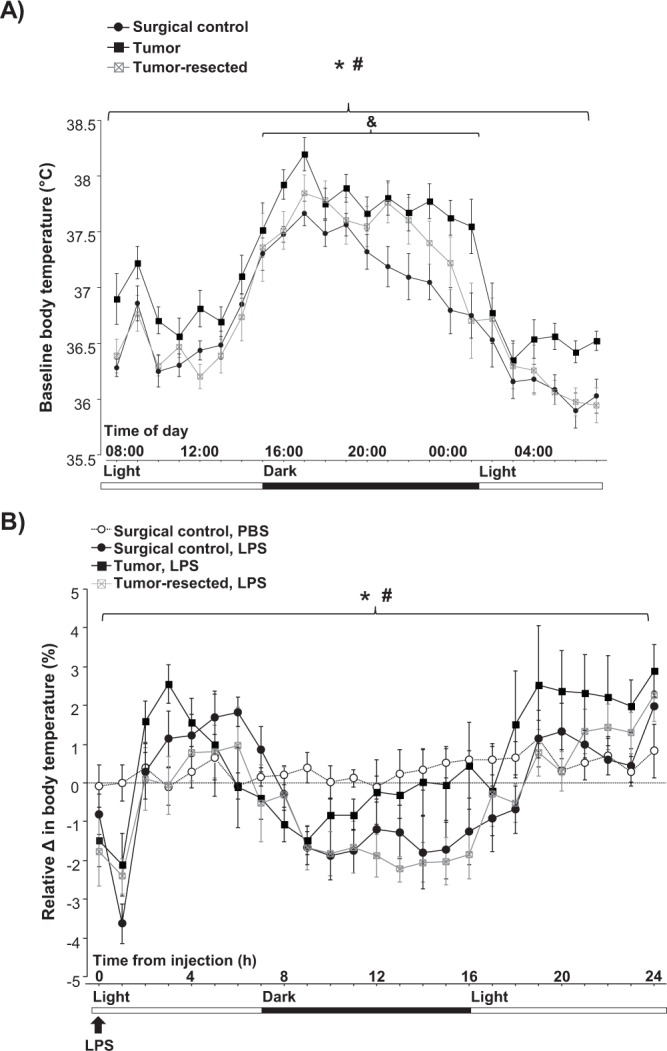
Tumor resection partially restores tumor-induced increases in basal body temperature and restores hyperthermic responses after LPS injection. Mean (±SEM) baseline body temperature 24 h before LPS injection (**A**) and relative body temperature change in response to LPS injection (**B**). *p ≤ 0.05 tumor bearing relative to surgical control mice injected with LPS; ^#^*p* ≤ 0.05 tumor-bearing relative to –resected mice injected with LPS; ^&^*p* ≤ 0.05 tumor-resected relative to surgical control mice injected with LPS by repeated measures ANOVA (n = 7–8 mice/group).

For body temperature before LPS treatment, repeated measures ANOVA revealed an effect of tumor treatment (*F*_2,27_ = 4.3, *p* < 0.05): baseline body temperature was elevated in tumor-bearing mice relative to tumor-free mice and tumor-resected mice during both the light and dark phases (*p* < 0.0001 in all cases; Fig. 4A). Tumor-resection reversed the tumor-induced increase in basal body temperature relative to tumor-free mice only during the light phase; body temperature remained elevated in tumor-resected mice relative to tumor-free mice during the dark phase (tumor treatment effect: *F*_2,27_ = 4.1, *p* = 0.005).

To control for differing baseline body temperature among tumor treatments, data after LPS were analyzed as a percent change from baseline (Fig. 4B). LPS injection induced percent changes in body temperature over time relative to PBS-treated controls (*p* < 0.0001 in all cases), causing fever during the light phase and hypothermia during the dark phase in tumor-free control mice. Of note, LPS injection acutely decreased body temperature for tumor-free and –resected groups during the first hour post injection (Fig. 4B; LPS main effect: *p* < 0.0005 and *p* < 0.005 respectively) and tended to do the same for tumor-bearing mice (*p* = 0.06). However, the kinetics of body temperature over the following hours differed among the tumor treatments groups. Among LPS-treated mice, a repeated measures ANOVA revealed that tumor and time interacted (*F*_2,18_ = 2.0, *p* < 0.05), such that tumors induced an increase in body temperature throughout the 24 h after LPS injection (tumor treatment effect: *F*_1,24_ = 1.6, *p* < 0.05) relative to tumor-free mice and, in part, an attenuation of LPS-induced hypothermia in the dark phase (Fig. 4B). During the initial light phase (2–8 h post-LPS), hyperthermia peaked earlier (3 h post-LPS) in tumor-bearing mice than in surgical controls (5 h post-LPS). Furthermore, tumor resection attenuated this hyperthermic period relative to surgical controls (tumor treatment effect: *F*_2,120_ = 2.1, *p* < *0*.*05*). The LPS-induced changes in body temperature receded 17 h after injection (beginning of the light phase) in control and tumor-resected mice, but body temperature remained increased for tumor-bearing mice relative to tumor-free (tumor treatment effect: *p* < *0*.*005* for PBS and *p* < *0*.*0005* for LPS-treated) and -resected mice (tumor treatment effect: *p* < *0*.*005*).

### Tissue masses

Tumor masses at the time of resection or final tissue collection were comparable (*p* > 0.05; Supplementary Fig. 1A), indicating that tumor-bearing and tumor-resected mice were exposed to comparable tumor burdens. LPS progressively increased relative spleen mass compared to PBS (LPS main effect: *F*_2,45_ = 9.3, *p* < 0.001; Supplementary Fig. 1B), such that spleens weighed more 24 h after LPS than 4 h after LPS (time main effect: *F*_1,39_ = 5.1, *p* < 0.05). Tumor treatments did not statistically significantly alter spleen mass post-LPS (*p* > 0.05), although tumors tended to augment splenomegaly 4 h post-LPS (*p* = 0.1).

### Tumor resection reverses late circulating cytokine responses to LPS

**Figure 5:**
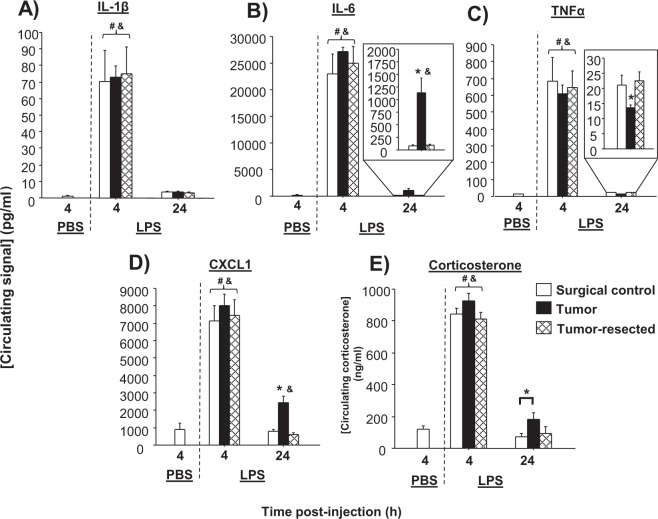
Tumors alter circulating cytokine and corticosterone responses to LPS. Mean ± SEM (**A**) IL-1β, (**B**) IL-6, (**C**) TNF-α, (**D**) CXCL1 (pg/ml), (**E**) corticosterone (ng/ml) in surgical controls, tumor-bearing mice, and tumor-resected mice (n = 10–20/group); ^&^*p* < 0.05 relative to PBS-treated mice; ^#^*p* < 0.05 relative to 24 h; *p < 0.05 relative to other treatments at the same time point.

Among LPS-treated mice, time influenced all four circulating cytokines examined, such that they were all elevated at 4 h post-injection relative to 24 h post-injection (IL-1β: *F*_1,60_ = 85.3, *p* < 0.0001; IL-6: F_1,60_ = 294.8, *p* < 0.0001; TNF-α: *F*_1,60_ = 142.3, *p* < 0.0001; CXCL1: *F*_1,60_ = 200.4, *p* < 0.0001; Fig. 5A). Tumor treatment independently increased overall CXCL1 (*F*_2,60_ = 3.7, *p* < 0.05), primarily driven by the 24-h post-LPS time point. Tumor treatment did not significantly affect overall cytokine concentrations for the other cytokines examined. However, based on our *a priori* hypothesis^4^, tumor-dependent changes in circulating IL-6 and TNF-α from the LPS treatment were observed 24 h post-injection. Specifically, recovery of both IL-6 (tumor treatment effect: *F*_2,34_ = 11.8, *p* < 0.0001; Fig. 5B) and CXCL1 (tumor treatment effect: *F*_2,34_ = 17.1, *p* < 0.0001; Fig. 5D) was attenuated for tumor-bearing mice relative to both tumor-free and -resected mice. In contrast, tumors precipitated the recovery of TNF-α after LPS relative to tumor-free and -resected mice (tumor treatment effect: *F*_2,34_ = 3.7, *p* < 0.05; Fig. 5C).

### Tumor-resection reverses the delayed circulating corticosterone recovery from LPS

Among LPS-treated mice, both time (*F*_1,43_ = 102.7, *p* < 0.0001) and tumor treatment (*F*_2,43_ = 3.7, *p* < 0.05) independently influenced circulating corticosterone concentrations (Fig. 5E). These overall differences were manifested at 4 h post-LPS by slightly higher corticosterone concentrations in tumor-bearing, relative to tumor-resected mice (*p* = 0.09), and higher corticosterone in tumor-bearing mice relative to controls at 24 h post-LPS (*p* ≤ 0.05). At 24 h post-LPS, corticosterone concentrations from all mice were comparable to PBS-injected controls (*p* > 0.05).

### Tumors impair neuroinflammatory responses to LPS; resection partially restores

#### Il-1β

**Figure 6:**
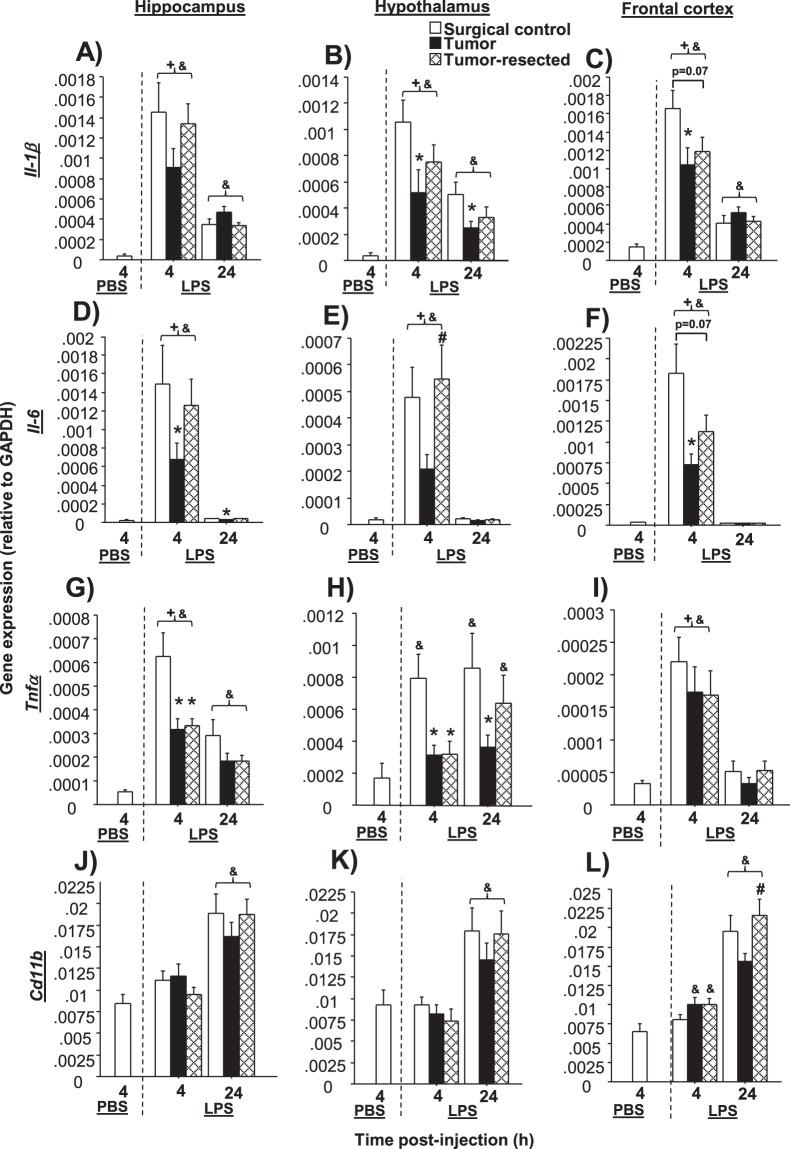
Effects of mammary tumors and tumor resection on mRNA expression of inflammatory markers in brain regions that regulate sickness behavior. Mean ± SEM TaqMan quantitative gene expression of *Il*-*1β* (**A**–**C**), *Il*-*6* (**D**–**F**), *Tnf*-*α* (**G**–**I**) and *Cd11b* (**J**–**L**) in the hippocampus, hypothalamus and frontal cortex in surgical controls, tumor-bearing mice, and tumor-resected mice (n = 10–20/group); *p < 0.05 relative to LPS-treated tumor-free mice at the same time point; ^#^*p* < 0.05 relative to tumor-bearing mice at the same time point; ^&^*p* < 0.05 relative to PBS-treated mice; ^+^*p* < 0.05 relative to 24 h.

LPS significantly increased *Il*-*1β* gene expression at both 4 and 24 h post-injection for all treatment groups relative to PBS-treated controls (*p* < 0.05 in all cases). Within LPS-treated groups, time since LPS injection influenced hippocampal (*F*_1,87_ = 37.8, *p* < 0.001) and frontal cortex *Il*-*1β* (*F*_1,87_ = 60.1, *p* < 0.0001; Fig. 6A–C), such that *Il*-*1β* levels receded over time after LPS injection for all tumor treatments. Tumor and time also interacted for hippocampal (*F*_2,87_ = 2.3, *p* < 0.05) and frontal cortex *Il*-*1β* expression (*F*_2,87_ = 3.9, *p* ≤ 0.05). Both time (*F*_1,88_ = 18.3, *p* < 0.0001) and tumor treatment (*F*_2,88_ = 5.5, *p* < 0.01) independently influenced hypothalamic *Il*-*1β* mRNA, which in contrast to our *a priori* hypothesis, was reduced in both the hypothalamus (*t*_27_ = 2.2, *p* < 0.05) and frontal cortex (*t*_30_ = 2.3, *p* < 0.05) of tumor-bearing mice relative to tumor-free controls at 4 h post-LPS. In the frontal cortex, tumor resection tended to reduce *Il*-*1β* at 4 h post-LPS relative to controls, similar to mice that retained their tumors (*p* = 0.07). At 24 h post-LPS, *Il*-*1β* remained significantly decreased in the hypothalamus in tumor-bearing mice relative to tumor-free controls (*t*_30_ = 2.7, *p* < 0.05), whereas tumor-resected responses remained intermediate.

#### Il-6

LPS significantly increased *Il*-*6* gene expression at 4 h post-injection for all tumor treatment groups relative to PBS-injected controls (*p* < 0.05 in all cases). At 24 h post-LPS injection, *Il*-*6* remained elevated in the hippocampus only in the surgical control group (*U* = 0, *p* < 0.001) compared to PBS-treated controls. *Il*-*6* responses in both the frontal cortex and hypothalamus had resolved in all LPS-treated groups at 24 h post-LPS injection (*p* > 0.05 in all cases).

Within LPS-treated groups, both time (hypo: *F*_1,84_ = 48.4, *p* < 0.0001; frontal cortex: *F*_1,87_ = 70.4, *p* < 0.0001) and tumor treatment (hypo: *F*_2,84_ = 3.8, *p* < 0.05; frontal cortex: *F*_2,87_ = 4.9, *p* < 0.01) independently influenced hypothalamic and frontal cortex *Il*-*6*, as well as their interaction (hypo: *F*_2,84_ = 3.6, *p* < 0.05; frontal cortex: *F*_2,87_ = 4.9, *p* < 0.01). *Il*-*6* mRNA levels receded over time since LPS treatment in all brain regions examined (*F*_1,87_ = 38.3, *p* < 0.0001 in each region; Fig. 6D–F). Hippocampal (*U* = 75, *p* < 0.05) and frontal cortex (*U* = 48, *p* < 0.005) *Il*-*6* responses 4 h post-LPS were reduced in tumor-bearing mice relative to tumor-free controls; a similar trend was observed in the hypothalamus (*p* = 0.06). Tumor resection resolved these tumor-induced decreases in *Il*-*6*, except in the frontal cortex, where resection tended to decrease *Il*-*6*, much like that of tumor-bearing mice (*p* = 0.07). At 24 h post-LPS injection, hippocampal *Il*-*6* remained reduced in tumor-bearing mice relative to tumor-free controls (*t*_27_ = 2.6, *p* < 0.05). No 24-h differences among tumor treatments were observed for *Il*-*6* in the hypothalamus or frontal cortex.

#### Tnf-α

LPS treatment significantly increased *Tnf*-*α* gene expression in the hippocampus and frontal cortex at 4 h post-injection for all tumor treatment groups relative to PBS controls (*p* < 0.05 in all cases); in the hypothalamus, *Tnf*-*α* was only increased in the surgical control group (*U* = 14.5, *p* < 0.005). At 24 h post-injection, *Tnf*-*α* remained elevated in the hippocampus of all LPS-treated groups relative to PBS-treated controls (*t*_53_ = 9.0, *p* > 0.005), but *Tnf*-*α* in the hypothalamus was elevated only in the surgical control (*U* = 19, *p* < 0.05) and tumor-resect (*U* = 54, *p* < 0.05) groups. *Tnf*-*α* was not elevated in the frontal cortex (*p* > 0.05) at this time point.

Among LPS-treated groups, hippocampal *Tnf*-*α* mRNA was influenced independently by time (*F*_1,87_ = 20.1, *p* < 0.0001) and tumor treatment (*F*_2,87_ = 8.4, *p* < 0.0005; Fig. 6G). Hypothalamic *Tnf*-*α* was influenced by tumor treatment alone (*F*_2,88_ = 6.5, *p* < 0.005; Fig. 6H), whereas time influenced frontal cortex *Tnf*-*α* (*F*_1,87_ = 37.5, *p* < 0.0001; Fig. 6I). Both tumors and tumor resection significantly reduced hippocampal (*F*_2,44_ = 6.7, *p* < 0.005) and hypothalamic (*F*_2,41_ = 7.0, *p* < 0.005) *Tnf*-*α* responses to LPS at 4 h post-injection relative to tumor-free controls, whereas this pattern was absent in the frontal cortex (*p* > 0.05). No *Tnf*-*α* differences among tumor treatments were observed in the hippocampus, hypothalamus, or frontal cortex 24 h post-LPS injection.

#### Cd11b

LPS treatment had no effect on *Cd11b* expression 4 h post-injection relative to PBS-treated controls in the hippocampus or hypothalamus (*p* > 0.05), but was elevated in the frontal cortex, specifically for the tumor and tumor-resected groups (tumor treatment effect: *p* < 0.05 in both cases). In contrast, LPS significantly increased *Cd11b* gene expression throughout the brain 24 h post-injection for all tumor treatment groups relative to PBS controls (*p* < 0.05 in all cases).

Within LPS-treated groups, hippocampal (*F*_1,93_ = 32.3, *p* < 0.0001; Fig. 6J), hypothalamic (*F*_1,88_ = 26.2, *p* < 0.0001; Fig. 6K) and frontal cortex (*F*_1,93_ = 61.9, *p* < 0.0001; Fig. 6L) *Cd11b* mRNA were all influenced by time. Differences among tumor treatments in *Cd11b* responses to LPS 4 h after LPS injection were absent in the hippocampus and hypothalamus. At 24 h post-LPS, *Cd11b* was comparable among tumor treatments in the hippocampus and hypothalamus. In the frontal cortex, tumor-bearing had reduced *Cd11b* expression relative to tumor-resected mice (*U* = 101, *p* < 0.05).

The relationships among tumor mass and neuroinflammatory responses were investigated using correlational analyses for both the tumor-bearing and tumor-resected mice. At 4 h post-LPS, current tumor mass predicted microglial marker *Cd11b* gene expression in all three brain regions examined (*p* < 0.05 in each case; Supplementary Fig. 2A), such that larger tumors resulted in greater *Cd11b* gene expression. Among tumor-resected mice, prior tumor mass predicted the resolution of neuroinflammation 24 h post-LPS, such that greater prior tumor mass was negatively correlated with *Tnf*-*α* and *Cd11b* responses, akin to tumor-bearing responses at this time point. This negative correlation between tumor mass and neuroinflammation 24 h post-LPS was statistically significant for hippocampal and hypothalamic *Tnf*-*α* and hypothalamic *Cd11b* (*p* ≤ 0.05 in each case; Supplementary Fig. 2B). While *Cd11b* mRNA did not mirror cytokine gene expression responses to LPS, understanding the effects of tumor treatments on the activation or polarization of microglia^23^ require further investigation with additional microglial markers. However, *Cd11b* expression in the brain during peak LPS response (4 h) was related to tumor burden, indicating that *Cd11b* may be influenced by the presence of a tumor during an inflammatory event. The correlations observed in the current study might potentially be associated with the inflammatory profile of the microglia/monocytes expressing *Cd11b*, since this marker does not decrease in tumor and tumor-resected mice, independently of the neuroinflammatory status and warrant future investigations. The correlations between all other genes and tumor mass were not statistically significant at 4 or 24 h post-injection (*p* > 0.05).

## Discussion

In the present study, using rodent models of breast cancer patients and survivors, peripheral tumors consistently attenuated neuroinflammatory gene expression and associated sickness behavior in response to an LPS peripheral immune challenge. These remarkable effects of tumors were primarily reversed by tumor removal, although select neuroinflammatory responses to LPS and basal body temperature changes persisted. The latter findings extend our previous observations that tumor removal partially reverses the basal inflammatory and physiological consequences of tumors^5^. Although two prior studies report that peripheral tumors exacerbate peripheral and central inflammatory responses, weight loss, and lethargy to an LPS injection^4,8^, the present conflicting data indicate that tumor type, species, and strain significantly modulates these responses. Notably, the present findings were derived from four treatment-balanced experiments, validating the significant repeatability of the results. Taken together, the basic science literature regarding the extent to which peripheral tumors can induce changes in the activity of innate peripheral and central immune pathways to influence behavioral changes remains relatively scarce and conflicting. Of note, neutropenia and lymphopenia are common side effects of cancer treatment (e.g., chemotherapy) which have immunosuppressive consequences (as well as inflammation), but treatments were absent in the current study. Furthermore, tumors play a dual role in modulating immune function, depending on the arm of the immune system (innate/inflammation vs. adaptive) and the stage and type of tumor^24^. In the clinical and preclinical literature, tumor-induced behavioral changes are most often correlated with increases in pro-inflammatory cytokines, which we reviewed in^25^. Indeed, myeloid-derived suppressor cells (MDSCs) are increased in the primary tumor site, as well as in the spleen and blood in this model, which increase peripheral and central pro-inflammatory cytokines and decrease T-cell function^5^.

Despite the reduced neuroinflammatory cytokine mRNA responses of the tumor group, LPS injection induced anorexia and decreased body mass to the same extent for all treatments (surgical controls, tumor-bearing, tumor-resected). Similarly, previous research using different approaches to evaluate neuroinflammatory signaling demonstrates that suppressed neuroinflammation does not prevent LPS-induced decreases in body mass or food intake after an immune challenge^26^. One potential explanation may be the relatively insensitive tools used to measure these sickness behaviors. For example, manual food intake measurement may omit small pieces of chow that have fallen into the bedding. In addition, a recent study implicates a nontraditional neuroinflammatory pathway as a key mediator of LPS-induced anorexia and weight loss, as well as in pancreatic cancer cachexia^27^. Genetic deletion of the adaptor protein TIR-domain-containing adaptor inducing interferon-β (TRIF) decreases microglial activation and attenuates anorexia and weight loss. Thus, the canonical inflammatory players examined in this study may be less important for these particular sickness behaviors.

In contrast, tumors significantly modulated minute-by-minute thermoregulatory and locomotor activity responses to LPS in the present study. Indeed, tumors altered body temperature both before and after the acute immune challenge relative to tumor-free mice. The observed elevations in baseline body temperature of tumor-bearing mice are comparable to previous findings in rodent tumor models and cancer patients^28–30^, and may, in fact, reflect an underlying disruption in circadian rhythm circuitry or temperature homeostasis. Notably, tumor resection was not able to reverse the tumor-induced disruption in body temperature regulation under basal conditions during the dark (active) phase. Thus, mammary tumor biology may have lasting effects on homeostatic pathways which persist >2 weeks after complete tumor resection. Indeed, among breast cancer survivors, thermal discomfort symptoms, such as hot flashes and night sweats, are highly prevalent (65–85%)^31–34^, although cancer therapies also likely contribute to these thermoregulatory changes.

The LPS-induced thermoregulatory response in mice is multiphasic and involves febrile and hypothermic phases with distinct physiological mechanisms and adaptive value^35^. In the present study, tumor-bearing mice exhibited a transient phase of fever, but failed to mount the hypothermic response observed in LPS-treated controls during the dark (active) phase of the daily light cycle. The observed suppressed neuroinflammatory response likely contributed to this impaired thermoregulatory response, as central proinflammatory cytokines (IL-1β, IL-6 and TNF-α) regulate LPS-induced fever and hypothermia^36,37^. For example, central administration of IL-6 restores the thermoregulatory response to LPS or IL-1β injection in IL-6-deficient mice^38^. Of note, our cancer-related findings are similar to the impaired sickness response (e.g., suppressed thermoregulatory response and decreased peripheral proinflammatory cytokines) observed in colitic rats after an LPS immune challenge^39^. Taken together, peripheral chronic inflammation, like tumors or colitis, may cause a compensatory inhibition of subsequent neuroinflammatory responses via humoral or neural inflammatory signaling pathways^39^. In tumor-resected mice, body temperature responses to LPS returned to control levels, demonstrating the causal role of the peripheral tumor. However, the hyperthermic responses to LPS remained partially impaired in the tumor-resected mice, but only during the light phase. Overall, these temporally-biased results suggest a potential disruption in circadian rhythmicity with tumor and tumor resection and warrant further investigation.

The moderate disruption in thermal response of tumor-bearing mice was accompanied by markedly decreased hippocampal and hypothalamic *Tnf*-*α* mRNA levels 4 h after LPS injection relative to tumor-free mice. Indeed, in a cancer cachexia rodent model, anti-TNF-α treatment partially reverses tumor-induced decreases in body temperature^40^. However, this robust decrease in *Tnf*-*α* persisted in tumor-resected mice without obvious consequences for body temperature responses to LPS. Thus, TNF-α is unlikely to be the sole mediator of thermoregulatory differences in these models.

Indeed, convergent lines of evidence indicate that various other pathways are critical for fever and hypothermic responses in immune-challenged mice^35,39,41^. Specifically, neuroendocrine pathways, such as the hypothalamic-pituitary-adrenal (HPA) axis and neurotransmitter systems, modulate several aspects of both responses (reviewed by^42^). Here, elevated circulating corticosterone concentrations were observed in tumor-bearing mice relative to tumor-free and -resected mice 24 h after immune challenge. This altered recovery in the corticosterone response to LPS may, therefore, be related to the coincident sickness behavioral changes observed in tumor-bearing mice. Indeed, chronic unpredictable stress in rats increases basal body temperature and hypothermic responses to a cold room challenge, relative to stress-free controls. Treatment with a corticosterone synthesis inhibitor resolves this increased cold challenge thermoregulatory response, although it does not attenuate the elevation in basal body temperature^41^.

As expected, LPS injection reduced locomotor activity in all treatments relative to the PBS-treated mice. However, the relatively attenuated magnitude of LPS-induced lethargy in tumor-bearing mice, compared to LPS-treated tumor-free and -resected mice, is consistent with their attenuated thermoregulatory and neuroinflammatory responses^43^. Indeed, a caspase-1 inhibitor centrally administered to rats, which significantly reduces the brain concentration of IL-1β (pre-frontal cortex, hypothalamus, and hippocampus), attenuates LPS-induced lethargy^44^. Our data contrast with previous work showing exacerbated deficits in locomotor activity in human papilloma virus (HPV)-related head and neck tumor-bearing mice 24 h after an LPS-induced challenge^8^. Although these discrepancies might reflect differences in the tumor model and in the methods used, the locomotor activity assessment in the current study was more sensitive and reliable (data collected every minute for 48 h) than the single 5-min open field test used to assess locomotion in the head and neck tumor model. Overall, more details about the mechanisms by which tumors alter thermoregulatory and other immune responses are warranted.

While prior studies suggest that peripheral tumors might prime the microglia and exacerbate neuroinflammatory responses to subsequent innate peripheral immune challenges^4,8^, our current data indicate that some peripheral tumor models, in fact, attenuate the LPS-induced gene expression of proinflammatory cytokines in the brain. Additionally, in this study, tumor mass positively predicted *Cd11b* microglial gene expression from brain tissue collected 4 h post-LPS in mice that retained their tumors, even though absolute values of this marker were not statistically different among tumor treatments at this time. This consistent correlation among various brain regions suggests a potential relationship between early microglial activation/number and tumor burden. Furthermore, former tumor mass of tumor-resected mice negatively predicted *Tnf*-*α* and *Cd11b* 24 h post-LPS, indicating that larger former tumor masses predicted more tumor-like (attenuated) later neuroinflammatory responses (24 h post-LPS injection). Therefore, tumor pathology in breast cancer survivors may help predict altered inflammatory signaling of activated pathways and associated behavior during survivorhood.

Indeed, the reversal of the majority of the tumor-induced behavioral and physiological responses to LPS by tumor resection suggests that cancer treatments and/or stress (and their interactions with tumor biology) may play key roles in the lasting behavioral consequences of cancer associated with inflammation^45^. Systematic investigation into these interactions is necessary. All mice received some type of surgery two weeks prior to the LPS challenge, indicating that wound healing or anesthetic exposure do not explain differences among treatment groups. Tumor-resected mice and sham controls received an additional earlier surgical experience (sham or tumor inoculation). However, the instances in which tumor-resected mice were more comparable to tumor-bearing mice than controls (*Tnf*-*α* and basal body temperature) indicate that these changes were likely due to tumors and not the number of prior surgical experiences.

In addition, the tumor model used in the current study is non-metastatic and did not induce overt cachexia or sickness behaviors during the tumor development, which could have confounded the evaluation of the sickness response to the immune challenge. Finally, besides the estrous cycle was not tracked in the present study, recent evidence indicates that male and female rodents exhibit comparable variability in a broad range of physiological and behavioral traits^46,47^, regardless of the estrous phase. Taken together, our findings support the hypothesis that tumors decrease the amplitude of the baseline body temperature diurnal rhythm, as well as attenuate neuroinflammation and associated sickness behavioral responses to subsequent innate immune challenges, and that tumor resection reverses most, but not all of these responses. Thus, these data suggest that cancer patients and survivors may be predisposed to some impairment of host defense responses against secondary diseases or challenges (e.g., surgery, infection, stressors). Although several studies corroborate the hypothesis that peripheral solid tumors are sufficient to induce baseline behavioral and neuroinflammatory changes in rodent tumor models (reviewed by^25^), very little research had focused on how cancer may affect these pathways upon activation. Sickness behaviors in response to an immune challenge are an important strategy for the survival of infectious diseases, and both exacerbated or decreased neuroinflammatory responses may be maladaptive^48–50^. Indeed, inflammatory pathways are also related to psychiatric symptoms in cancer patients^25^, and therefore, alterations in pathway responses may have implications for behavioral comorbidities beyond sickness behavior. A better understanding of the mechanisms underlying these complex tumor-induced changes may provide insight into therapeutic targets and alternative treatments that allow for better health outcomes and quality-of-life for cancer survivors.

## Methods

### Animals

Nulliparous female 8- to 9-week old Balb/c mice (Charles River, Wilmington, MA, USA; see Fig. 1 for samples size) were single-housed and acclimated to the temperature-controlled (22 ± 1 °C) vivarium for 1 week under a 14:10 light:dark cycle (lights off at 015:00 h). Rodent chow (Harlan 7912) and water were available *ad libitum* throughout the study and cotton nestlets and plastic huts were provided for nesting. All animal experiments were approved by the Ohio State University Institutional Animal Care and Use Committees and carried out in accordance with the National Institutes of Health Guide for the Care and Use of Laboratory Animals (NRC, 2011). All efforts were made to minimize animal suffering and to reduce the number of mice used. This project consisted of four treatment-balanced experimental replications (two for neuroinflammation, two for emitter behavior).

### Cells

The murine mammary non-metastatic 67NR cancer cell line, originating from a spontaneous mammary adenocarcinoma in a Balb/c mouse^51,52^, was generously provided by Drs. Fred Miller and Lisa Polin at the Karmanos Cancer Institute. Cells were grown in DMEM with 10% FBS, 2 mM _L_-glutamine, 1 mM non-essential amino acids at 37 °C with 5% CO_2_.

### Tumor Induction

Prior to and after tumor induction, mice were acclimated to handling twice/week (n = 108). Under anesthetization (isoflurane vapors), a 5 mm incision was made medial to the 4^th^ nipple, 5 × 10^6^ cells (in matrigel) were injected into the 4^th^ mammary fat pad, and incisions were closed with a wound clip^5^. This procedure results in an *in situ* primary mammary carcinoma^53^ that does not metastasize^54^. This is a validated orthotopic and syngeneic breast cancer model, eliminating the need to use immunocompromised mice^5^. Mice assigned to the tumor resection group (n = 53) were inoculated with tumor cells 2 weeks prior to the other groups, so that the timing of their tumor resection surgeries corresponded to the time of tumor/control induction surgeries in the other groups. This allowed for simultaneous behavioral and physiological assessments, as well as the same interval between surgery and behavioral testing for all groups. Ear notches were made at tumor inoculation for individual identification purposes. Body mass was measured twice/week. Tumor induction was unsuccessful in 9 mice and they were removed from the analyses.

### Tumor resection

A modified radical mastectomy procedure was used to completely remove primary tumors in the “survivor” group^5^. These mice were anesthetized (isoflurane), and tumors with intact membranes were surgically removed along with mammary tissue, fat, and lymph nodes. Skin was closed with wound clips. Buprenorphine (0.05 mg/kg; s.c.) was administered immediately after surgery and again 12 h later. Complete tumor resections were verified at necropsy, and any mice with recurring tumors (n = 8) were removed from the study. The surgical controls were given a sham resection surgery at this time.

### Telemetry

A subset of mice was singly-housed for a week and randomly assigned to the following experimental groups: (1) surgical control/PBS, (2) surgical control/LPS, (3) tumor/LPS, or (4) tumor resection “survivor” group/LPS. This subset was then implanted intraperitoneally with transmitters (G2 E-mitter; Starr Life Sciences, Oakmont, PA, USA) under isoflurane anesthesia. Buprenorphine (0.05 mg/kg; s.c.) was administered immediately after surgery and again every 4–6 h for 72 h post-surgery. Individual cages were placed on receiving platforms (ER-4000 receiver, Starr Life Sciences) connected to a computer that collected the body temperature and locomotor activity signals from the implanted transmitter every 1 min, which were then converted to a longitudinal record via Vital View software (v. 5.1, Mini-Mitter, Bend, OR, USA). In both series of treated-balanced experiments, there was one transmitter failure and the mice (n = 2) were removed from the body temperature and locomotion collection data.

### Acute inflammation induction

Mice were injected with bacterial lipopolysaccharide (*E*. *coli*, 026:B6, 250 μg/kg in 100 μl PBS, i.p.)^4,43,55^ at 08:00 h EST. Surgical control mice received 100 μl PBS.

### Sickness Behavior

Body mass, food intake, locomotion, and body temperature (the latter two from the implanted transmitters) were recorded for a subset of mice at 4 and/or 24 h after LPS injection. The baselines for these parameters were measured 24 h prior to injection.

### Tissue collection

Following rapid decapitation, blood was collected through heparin-lined natelson blood tubes, kept on ice and then centrifuged (20 min at 2,000 rpm), and plasma was stored at −80 °C until assayed for cytokine and corticosterone quantification. Brain regions relevant to sickness behaviors (hippocampus, hypothalamus, frontal cortex) were dissected out and frozen in RNA Later preservative for later neuroinflammatory gene expression assessment. Tumors and spleens were removed aseptically and weighed.

### Plasma cytokine concentrations

To compare systemic inflammation among treatments, the key LPS-induced pro-inflammatory cytokines relevant to sickness behavior interleukin (IL)-1β, IL-6, tumor necrosis factor (TNF-α)^4,56,57^, and keratinocyte chemoattractant (CXCL1), which is crucial in the innate immune response after an immune challenge^58^ and has previously demonstrated to be relevant to this tumor model^5^, were measured in plasma samples using a multiplex fluorescent bead array (Mouse Inflammatory 4-Plex Panel; Invitrogen, Frederick, MD, USA), following the manufacturer’s protocol.

### Plasma corticosterone concentrations

Glucocorticoids are potent innate anti-inflammatory agents and are produced in response to a peripheral LPS injection^59^. Corticosterone was measured in duplicate in plasma samples via EIA according to the manufacturer’s instructions (Enzo Life Sciences, Plymouth Meeting, PA, USA) after 1:40 dilution. The threshold of detection was 27 pg/ml. Intrassay and interassay variations were each <10%.

### Quantitative RT-PCR

Total RNA was extracted from the brain hippocampus, hypothalamus, and frontal cortex using Qiagen RNeasy mini kits (Valencia, CA, USA). RNA concentrations were measured and 260/280 ratios were determined to be 1.8–2.0 (NanoDrop, Wilmington, DE, USA). Total RNA was reverse transcribed using SuperScript First-Strand kits (Invitrogen, Grand Island, NY, USA), following the manufacturer’s protocol. Four genes of interest were chosen based on their established role in neuroinflammation-induced sickness behaviors (*Il*-*1β*, *Il*-*6*, *Tnf*-*α*, *Cd11b*)^4,55,60^. Mouse TaqMan Gene Expression Assays were purchased from Applied Biosystems (Carlsbad, CA, USA) with probes labeled with 6-FAM and MGB (non-fluorescent quencher) at the 5′ and 3′ ends, respectively: *Il*-*1β* (Mm00434228_m1), *Il*-*6* (Mm00446190_m1), *Tnf*-*α* (Mm00443258_m1), *Cd11b* (Mm00434455_m1), *Gapdh* (Mm99999915_g1; labeled with VIC). The following universal two-step RT-PCR cycling conditions on the 7000 Sequence Detection System (Applied Biosystems) were used: 50 °C (2 min), 95 °C (10 min), 40 cycles of 95 °C (15 s) and 60 °C (1 min). Relative gene expression of individual samples run in duplicate was calculated by the comparative C_T_ method (2^−ΔCT^).

### Statistical analysis

Statistical comparisons of body mass, food intake, tissue mass, gene expression, circulating cytokines and corticosterone were analyzed using 2-way ANOVAs followed by *post*-*hoc* Fisher’s LSD or Student’s *t*-tests with Statview version 5.0.1 software (Scientific Computing, Cary, NC, USA) when variance was normal. Nonparametric Mann-Whitney *U* tests were used when variance was not normally distributed. Repeated measures ANOVAs were used to compare changes in body temperature and activity responses over time. Pearson’s correlations were used to assess the relationships among variables. Due to differences in the baseline measures for body temperature between the surgical control and tumor/tumor-resect groups, the influence of LPS injection on temperature was calculated as a percent change relative to the baseline recordings (i.e., calculated within individual relative to the 24 h prior to the LPS injection). Data were determined to be statistically significant when *p* ≤ 0.05 and are presented as mean ± standard error of the mean (SEM).

## Supplementary information

Supplementary Dataset 1
