# Supplementary material for: Tumor resection ameliorates tumor-induced suppression of neuroinflammatory and behavioral responses to an immune challenge in a cancer survivor model
Source: Sci Rep. 2019 Jan 24;9:752. doi: 10.1038/s41598-018-37334-8 (PMC6345941; doi:10.1038/s41598-018-37334-8)
Supplement: Supplementary file 1 — Supplementary Dataset 1 [file 41598_2018_37334_MOESM1_ESM.pdf]

Tumor resection ameliorates tumor-induced suppression of neuroinflammatory and behavioral responses to an immune challenge in a cancer survivor model

Jessica C. Santos, Savannah R. Bever, Gabriela Pereira-da-Silva, Leah M. Pyter

SUPPLEMENTARY FIGURE 1

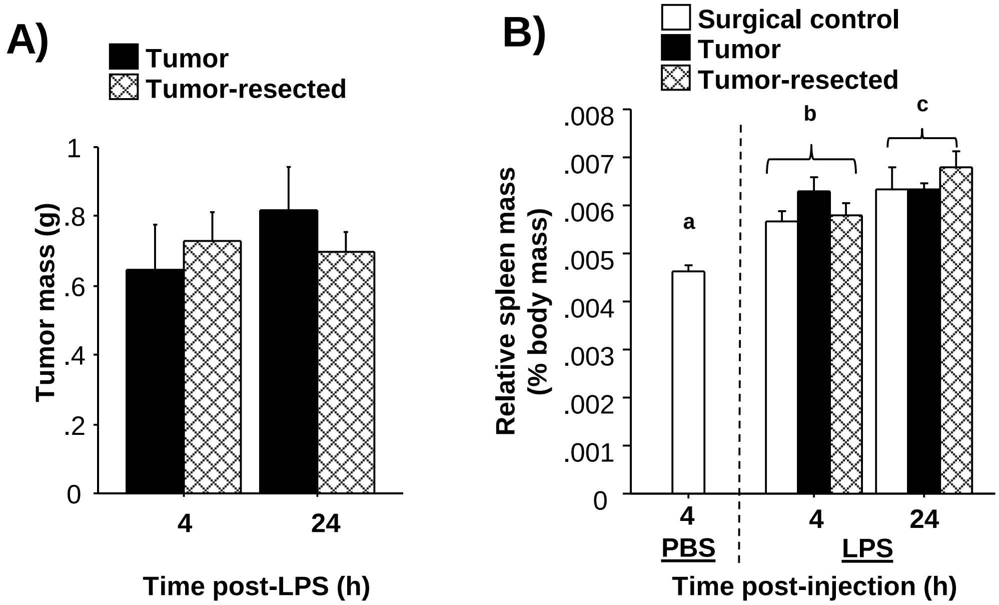

Supplementary Figure 1. Effects of complete mammary tumor resection on spleen masses. Mean ± SEM **(A)** tumor mass taken at surgical resection (tumor-resected) or final tissue collection (tumor), **(B)** spleen mass relative to body mass in PBS-treated, tumor-free controls or 4 or 24 h post-LPS among tumor treatments. n=7-20/group; letters represent statistical differences.

Tumor resection ameliorates tumor-induced suppression of neuroinflammatory and behavioral responses to an immune challenge in a cancer survivor model

Jessica C. Santos, Savannah R. Bever, Gabriela Pereira-da-Silva, Leah M. Pyter

SUPPLEMENTARY FIGURE 2

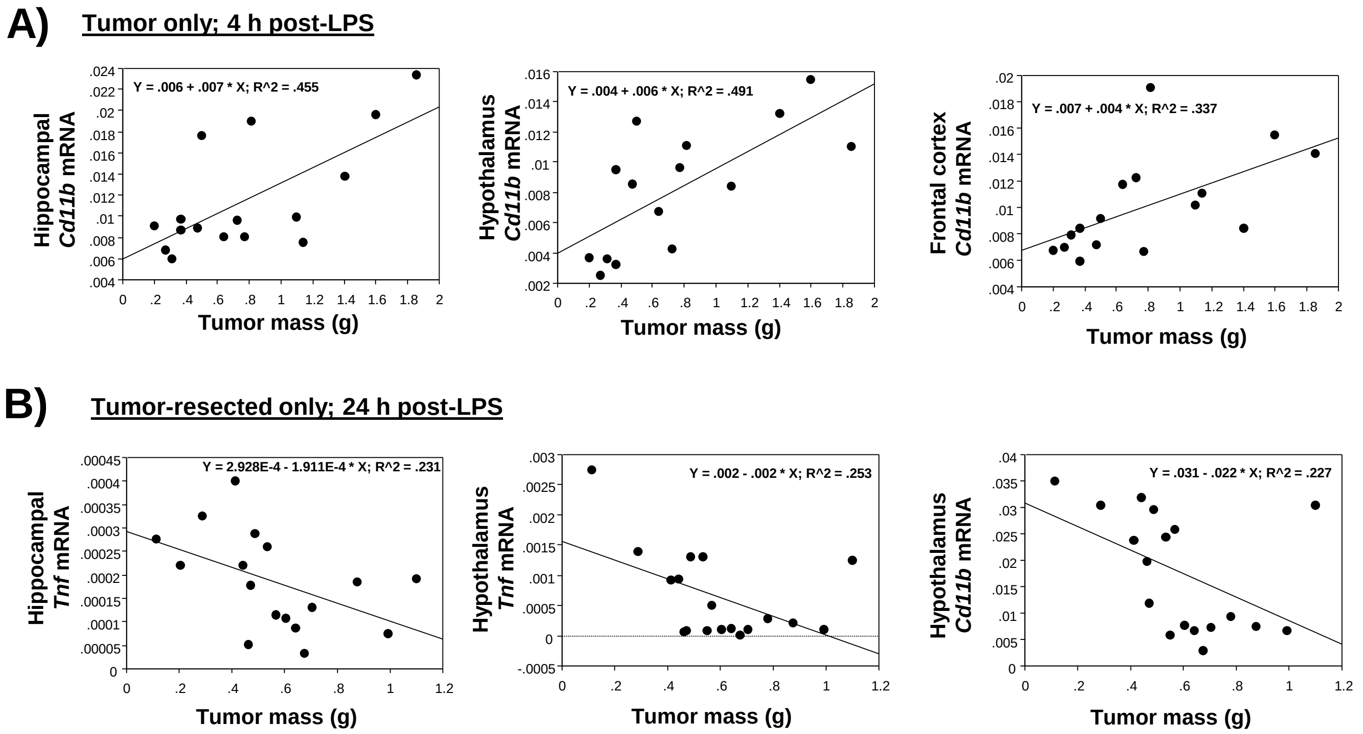

Supplementary Figure 2. Correlation between tumor mass and neuroinflammatory response to LPS challenge. Linear regressions between total mammary tumor mass, cortical and hippocampal *Cd11b* mRNA expression in tumor bearing mice 4 h after LPS injection **(A)**. Correlation between prior tumor mass and the resolution of neuroinflammation in the hippocampus and hypothalamus 24 h post-LPS **(B)**.
